# Synthesis of Highly Tunable Alloy Nanocatalyst through Heterogeneous Doping Method

**DOI:** 10.1002/advs.202204693

**Published:** 2022-12-12

**Authors:** Yong Beom Kim, Seunghyun Kim, Jinwook Kim, Jun Kyu Kim, Seung Jin Jeong, DongHwan Oh, WooChul Jung

**Affiliations:** ^1^ Department of Materials Science and Engineering Korea Advanced Institute of Science and Technology (KAIST) 291 Daehak‐ro, Yuseong‐gu Daejeon 34141 Republic of Korea; ^2^ Present address: Samsung Advanced Institute of Technology (SAIT) 130 Samsung‐ro, Yeongtonggu Suwon 16678 Republic of Korea; ^3^ Present address: Samsung Electronics 129, Samsung‐ro, Yeongtong‐gu Suwon 16677 Republic of Korea

**Keywords:** alloy, CeO_2_, CO oxidation, ex‐solution, grain boundary, heterogeneous doping, reverse water gas shift reaction

## Abstract

The combination of supported metal nanoparticles and functional host oxides catalyze many major industrial reactions. However, uniform dispersion and ideal chemical configuration of such nanoparticles, which determines the catalytic activity, are often difficult to achieve. In this study, a unique combination is proposed of heterogeneous doping and ex‐solution for the fabrication of Pt–Ni alloy nanoparticles on CeO_2_. By manipulating the reducing conditions, both the particle size and composition are precisely controlled, thereby achieving a highly dispersed and stable alloy nanocatalyst. The unique behavior of controlled alloy composition is elucidated through classical diffusion and precipitation kinetics with elemental analysis of the grain boundaries. Finally, Pt–Ni alloy nanocatalysts are successfully tuned showcasing a breakthrough performance compared to single element catalyst in reverse water gas shift reaction with superior stability and reproducibility.

## Graphical abstract

The heterogeneous doping method is reconceptualized for a rational design principle of alloy systems. Comprehensive interpretation on the composition variation of Pt–Ni alloy nanoparticles ensures its excellent tunability. Synthesized Pt–Ni alloy nanoparticles exceeds the performance of single element catalyst in reverse water gas shift reaction along with robust stability and reproducibility.

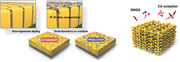

## 1 Introduction

Oxide‐supported metal nanoparticles have been widely studied as heterogeneous catalysts due to their high dispersion, highly undercoordinated sites, and metal‐support interaction.^[^ ^1^, ^2^, ^3^, ^4^, ^5^, ^6^, ^7^, ^8^, ^9^ ^]^ However, the degradation of particles at high operating temperatures is unavoidable, and such low thermal resistance limits their application.^[^ ^10^, ^11^, ^12^, ^13^ ^]^ Numerous studies in various areas have been conducted in an effort to hinder such phenomena by introducing a protective layer, such as a core–shell layer,^[^ ^14^, ^15^, ^16^ ^]^ an overcoating,^[^ ^17^, ^18^ ^]^ and others.^[^ ^19^, ^20^ ^]^ Recently, the “ex‐solution” approach, a particle synthesis method that relies on a surface phase transition of the support material, was introduced.^[^ ^21^, ^22^ ^]^ Through a reduction of reducible‐metal‐doped supports, the precipitation of secondary elements forms anchored metallic particles on the support, in turn stabilizing the nanosized particles from degrading in high‐temperature applications.^[^ ^23^, ^24^, ^25^, ^26^, ^27^, ^28^, ^29^, ^30^, ^31^, ^32^, ^33^ ^]^ For example, Neagu et al. synthesized La_0.4_Ca_0.4_Ni_0.03_Ti_0.97_O_3_‐supported Ni nanoparticles for coking‐resistant catalysts for use in methane reforming,^[^ ^34^ ^]^ and Nishihata et al. induced sintering‐resistant properties in Pd nanoparticles for use in catalytic converters.^[^ ^35^ ^]^ However, because the conventional ex‐solution process involves typical phase‐transition phenomena, the selection of both the doping element and the support is highly restricted such that a majority of studies on ex‐solution involve perovskite materials.^[^ ^36^, ^37^, ^38^, ^39^ ^]^

In this paper, we employ a method that combines heterogeneous doping and a grain boundary ex‐solution process.^[^ ^40^ ^]^ Compared to the conventional ex‐solution process, the proposed method utilizes the extrusion of elements doped in the grain boundaries instead of the bulk. By introducing a reducing atmosphere, metal in the grain boundaries evolves on the surface in the form of nanoparticles. This method holds various advantages due to its unique process. First, highly defective grain boundaries stimulate the rapid diffusion of the metal through the support oxide, which facilitates the overall process even at mild temperatures.^[^ ^41^, ^42^, ^43^, ^44^ ^]^ Second, the “diffusion” of the metal through the support grain boundaries allows elements to be equally supplied throughout the support, promoting the uniform dispersion of the nanoparticles. Third, metal nanoparticles ex‐solved on the surface are anchored in the grain boundaries and corners with enhanced thermal stability. Fourth, particle agglomeration can be recovered through subsequent oxidation and reduction steps due to the reversibility of the particle dissolution and evolution process. Finally, even metals with low bulk solubility levels can be stored in defective grain boundaries, allowing various combinations of metal and oxide support materials compared to the conventional ex‐solution method, where the choice of metal source is limited due to the requirement of doping in the bulk lattice.^[^ ^45^, ^46^ ^]^ Through this phenomena, precious metals such as Pt, which has high catalytic activity but very low lattice solubility, became applicable for use in heterogeneous catalysts.^[^ ^40^ ^]^

Despite such advantages, an in‐depth understanding of how the aforementioned phenomenon proceeds is lacking in multicomponent systems. Alloy nanoparticles exhibit properties superior to those of single‐metal nanoparticles, such as high catalytic activity, good chemical and physical stability, and various unprecedented properties.^[^ ^47^, ^48^, ^49^, ^50^, ^51^, ^52^ ^]^ Owing to such unique properties, these materials hold great promise for catalytic applications in renewable energy, petrochemistry, and in other chemical conversion reactions.^[^ ^23^, ^53^, ^54^, ^55^, ^56^, ^57^, ^58^ ^]^ Moreover, it is known that the configuration of the surface species, the geometry of the adsorption sites, and the electronic properties can be the origin of the superior catalytic activity, all of which can be tailored by altering the catalyst size and composition.^[^ ^51^, ^59^ ^]^ Therefore, methods that offer control of the size and composition of nanocatalysts can be considered essential for their use.

In this study, we applied heterogeneous doping method to create Pt–Ni alloy nanoparticles on CeO_2_. For a discrete analysis, we employed two classes of CeO_2_ as a support: a dense thin film for a detailed elucidation of the particle evolution behaviors and a columnar structure for a practical catalytic examination. A quantitative analysis using scanning electron microscope (SEM) and transmission electron microscope (TEM) revealed that an increase in the reduction temperature led to an increase in the particle size and a decrease in the particle density. Through STEM energy dispersive X‐ray spectroscopy (EDS), the increase in the Pt content in the Pt–Ni alloy particles was found to rely on the reduction time. Such interesting alloy composition variations were clarified through a time‐of‐flight secondary ion mass spectroscopy (ToF‐SIMS) elemental analysis and by classical diffusion and precipitation kinetics. The feasibility as a catalyst and incomparable performance to the single‐element catalyst were verified through reverse water gas shift and CO oxidation reaction.

## 2 Results and Discussion

### 2.1 Heterogeneous Doping Method

**Figure 1:**
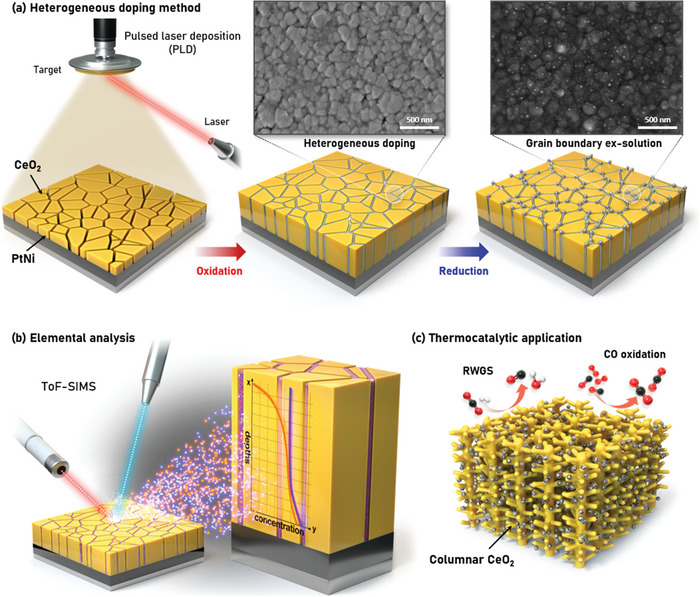
a) Schematic diagram of heterogeneous doping method. Oxidation was executed for in‐diffusion of the metal source into the grain boundaries and then reduction was performed for nanoparticle ex‐solution. Top‐view SEM image of CeO_2_ film after oxidation and subsequent reduction. b) Schematic of elemental analysis of Pt and Ni in the CeO_2_ grain boundaries through time‐of‐flight secondary ion mass spectrometry (ToF‐SIMS). c) Catalysis of various chemical reactions by introducing a columnar structured CeO_2_ deposited by PLD.

We prepared a stack of CeO_2_, Pt–Ni, and Al_2_O_3_ to analyze the particle evolution behavior in terms of the particle size and composition. Pt and Ni, the metal sources used here for heterogeneous doping, were sputtered onto an Al_2_O_3_ substrate and then annealed in order to ensure the alloying of these two metals. Then, 250 nm of CeO_2_ film was deposited via pulsed laser deposition (PLD) to ensure an ideal flat surface for the particle analysis. On the synthesized samples, Pt–Ni alloy nanoparticles were ex‐solved by a two‐step process, as shown in **Figure** **1**a, involving the heterogeneous doping of the metal source into the CeO_2_ grain boundaries and the ex‐solution of the metal nanoparticles onto the surface. During the heterogeneous doping step, the sample is oxidized at 700 °C for 10 h for sufficient metal diffusion throughout the CeO_2_. The metal layer underneath CeO_2_ is oxidized, and ionized metals diffuse into the grain boundaries of CeO_2_ (Figure S1, Supporting Information). We confirm that at 700 °C, the metal ions diffuse only through the grain boundary; as stated in Harrison's classification, when the process temperature is lower than one‐third of the support's melting temperature, which is 825 °C for CeO_2_ (*T*_m_ = 2475 °C), diffusion through the bulk lattice is negligible and grain boundary diffusion dominates.^[^ ^61^ ^]^ After the heterogeneous doping step, the metal is ex‐solved on the surface of the support through a subsequent reduction step. Because the metal source diffuses through the grain boundary, ex‐solved particles are anchored on the surface grain boundaries. During this step, we varied the reduction time and temperature to control the particle size and composition.

### 2.2 Characterization of the CeO_2_ Film and Metal Layer

In order to confirm the phase stability of CeO_2_ during the heat treatment, the synthesized sample was analyzed by XRD. As shown in Figure S2 (Supporting Information), crystallized CeO_2_ with randomly oriented grains and the metal source Pt–Ni alloy were visible. During the oxidizing and reducing heat treatments, CeO_2_ was stable and maintained its initial phase. The average grain size was calculated through the Scherrer equation and was 24.2±1.0 nm in diameter. The FWHM data used for calculation are shown in Figure S3 in the Supporting Information. The Pt–Ni alloy was distinguishable through a slight shift (0.06°) in the prominent peak compared to Pt (39.239°), as shown in Figure S4 (Supporting Information), where Pt–Ni and Pt were deposited on Al_2_O_3_ and annealed for the comparison. An FFT analysis was also conducted to verify the synthesized CeO_2_ support, as shown in Figure S5 in the Supporting Information. After each oxidation and reduction step, a top‐view SEM image was taken to characterize the deposited CeO_2_. The reduction process smoothed the grain's morphology but had a negligible effect on the grain size.

### 2.3 Effects of Reduction Conditions on the Particle Dispersion and Composition

**Figure 2:**
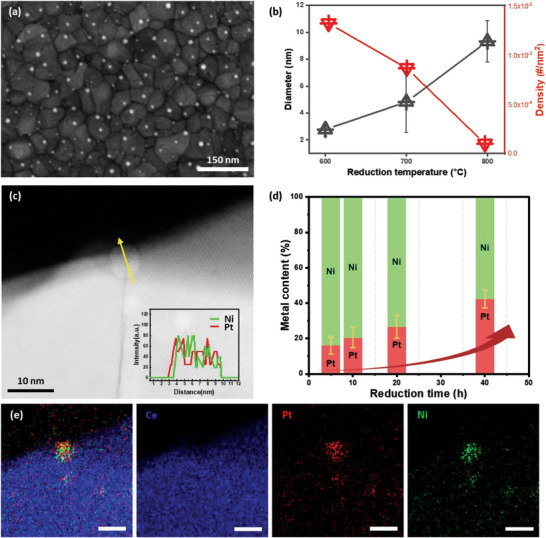
a) Surface SEM images of ex‐solved Pt–Ni nanoparticles on CeO_2_ films. b) Controlled size and particle density of Pt–Ni alloy nanoparticles ex‐solved on CeO_2_ after reduction at various temperatures. c) Cross‐sectional high‐angle annual dark‐field STEM (HAADF STEM) image of Pt–Ni alloy nanoparticle on the grain boundary of dense CeO_2_ film. The inset shows the energy dispersive X‐ray spectroscopy (EDS) line scan of the yellow arrow indicating the Pt–Ni alloy particle. d) Controlled composition of Pt–Ni alloy nanoparticles ex‐solved on dense CeO_2_ at various reduction time. e) EDS mapping images of Pt–Ni alloy nanoparticles after reduction at 700 °C for 20 h. (scale bars: 10 nm).

The reduction temperatures were varied at 600, 700, and 800 °C in order to control the size and density of the ex‐solved particles. All reduction processes lasted for 10 h and were done in an Ar‐balanced 4% H_2_ atmosphere. SEM images of the reduced samples are shown in **Figure** **2**a and Figure S6 in the Supporting Information. A quantitative analysis of the particle size and density was carried out using SEM. As the reduction temperature was increased, the density of the ex‐solved particles decreased and the size of the particles increased. These findings are in good agreement with the common behavior of ex‐solved particles and the nucleation and growth principles, where the rate for particle nucleation is higher at the low‐temperature regime, and the rate for particle growth is higher at the high‐temperature regime.^[^ ^61^, ^62^, ^63^ ^]^

An analysis of the composition of the ex‐solved Pt–Ni particles was carried out through STEM EDS, as shown in Figure 2d,e. TEM samples were created by cutting out a cross‐sectional layer perpendicular to the sample using the FIB method for a precise analysis of the particles ex‐solved on top of the support surface (Figure S7, Supporting Information). The reduction time was varied at 5, 10, 20, and 40 h at a reduction temperature of 700 °C. The Pt content of the ex‐solved alloy particles increased from 16 at% when reduced for 5 h to 41.7 at% for the samples reduced for 40 h. For every reduction condition, Pt and Ni were homogeneously mixed throughout the synthesized particles, forming a spherical, single‐phase particle. STEM and EDS mapping images of each sample are exhibited in Figure S8 in the Supporting Information.

**Figure 3:**
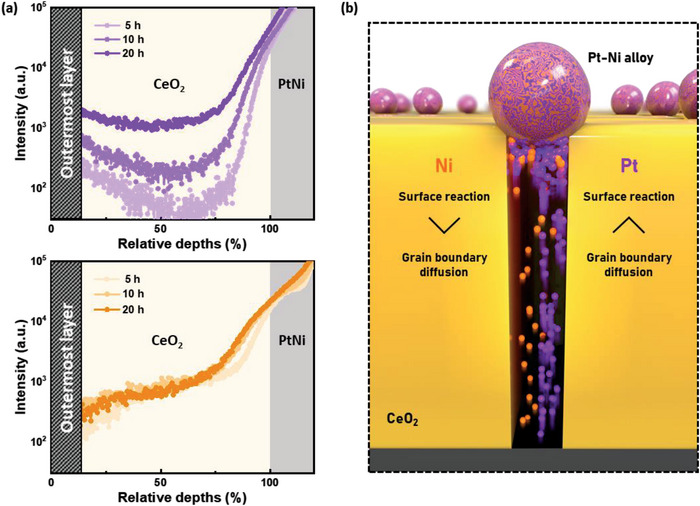
a) Concentration profiles of Pt and Ni in CeO_2_ grain boundaries acquired through ToF‐SIMS in terms of reduction time. b) Schematic of distinct precipitation kinetics of Pt and Ni in CeO_2_ grain boundaries. (Schematic was illustrated for improved understanding and does not represent the alloy uniformity).

The change in the alloy composition can be understood through the different diffusion and surface reaction kinetics of Pt and Ni in the CeO_2_ grain boundaries. Grain boundary ex‐solution can be seen as a two‐step process; the first step is the diffusion of the source elements to the surface of the support CeO_2_ and the second step is the surface reaction, which includes the nucleation and growth of the particles.^[^ ^64^, ^65^ ^]^ As in most reactions, the rate of the total grain boundary ex‐solution process is determined by the slowest step, referred to as the rate‐determining step (RDS). According to the classical precipitation kinetics, the depth profiles of the dopant elements in the CeO_2_ grain boundary differ depending on the RDS; when the surface reaction step is the limitation step, the concentration profile of the dopant shows a linear shape throughout the CeO_2_ layer, and when the diffusion step is the limitation step, the profile shows a decreasing concentration towards the surface of CeO_2_ (Note S1, Supporting Information). For each case, the rate of grain boundary ex‐solution, stated in terms of the flux, can be expressed as shown in Equations S1 and S2 (Supporting Information). In order to confirm the different RDSs of elements Pt and Ni for grain boundary ex‐solution, ToF‐SIMS was employed for depth profiling of the Pt and Ni contents in the CeO_2_ grain boundaries. The depth profiles for the assessment of the concentrations of Pt and Ni in the CeO_2_ grain boundaries are illustrated in **Figure** **3** in terms of the reduction time. The profile of Pt shows a linear shape in every sample regardless of the reduction time, meaning that the surface reaction is the RDS. In this case, the total flux of the grain boundary ex‐solution of Pt equals that of its flux for the surface reaction (Equation S1, Supporting Information). In contrast, the depth profile of Ni shows a decreasing trend towards the surface, which is proof that the RDS is the diffusion step of Ni in the grain boundaries. In this case, the flux of the grain boundary ex‐solution of Ni equals that of the corresponding flux for diffusion (Equation S2, Supporting information). Because the surface reaction of Pt is sluggish, as reduction time increases, the diffused Pt accumulates at the upper end of the grain boundaries, increasing its surface concentration. However, for Ni, no accumulation occurs due to its facile surface reaction compared to the diffusion in the grain boundaries, with its concentration profile thus maintained during the continuous reduction process. Given the results above, the increasing trend of the Pt composition in the ex‐solved particles can be explained through the change in the flux for grain boundary ex‐solution. Given that both the flux for the surface reaction and the flux for diffusion are proportional to the surface concentration of each species (Note S1, Supporting Information), the total flux of the grain boundary ex‐solution of Pt increases with the reduction time, whereas that of Ni remains unchanged, leading to a greater concentration of Pt in the particles after a longer reduction time.

### 2.4 Catalytic Performance

**Figure 4:**
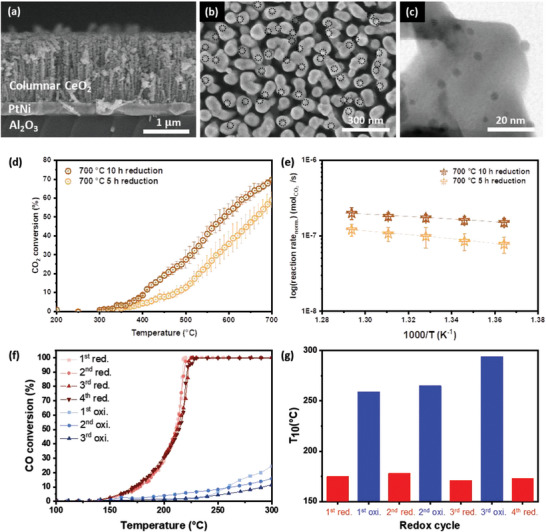
a) Cross‐sectional SEM image and b) surface SEM image of reduced sample. The dashed circles indicate ex‐solved Pt–Ni alloy particles. c) TEM image of Pt–Ni nanoparticles ex‐solved on columnar CeO_2_. d) Light‐off‐curve of Pt–Ni catalyst with different alloy composition (dark: reduced for 10 h, light: reduced for 5 h) for CO_2_ conversion and e) Arrhenius plot for reverse water gas shift (RWGS) reaction. f) Light‐off‐curve of CO oxidation and g) 10% CO conversion temperature (*T*_10_) of Pt–Ni/CeO_2_ after each redox step.

The aforementioned observations illustrate that the particle dispersion and composition can be controlled if using appropriate reducing conditions. Because the alloying of nanoparticles is known to alter the catalytic properties in a range of ways, viable evaluation methods of synthesized alloys are strongly desired in the field of heterogeneous catalysts. In the following section, we evaluate the catalytic activity of ex‐solved Pt–Ni nanoparticles with different compositions toward the reverse water gas shift reaction to ensure the feasibility of the catalytic process and the utility of the proposed tunability. Pt–Ni nanoparticles with different compositions were synthesized on columnar CeO_2_ by reducing each sample for 5 (sample name: R‐5) and 10 (sample name: R‐10) h, respectively (Figure S9, Supporting Information). The morphology of the synthesized catalyst is illustrated in **Figure** **4**a–c as determined via SEM and TEM, and the characterization data is listed in Table S1 in the Supporting Information. The catalytic performance was tested in a fixed‐bed flow reactor in a temperature range of 200–700 °C. The light‐off curve and the resulting Arrhenius plot are shown in Figure 4d,e, respectively. Catalyst R‐10 showed superior performance in the conversion of CO_2_ compared to R‐5 in the entire temperature range used for the measurement. At a reaction temperature of 480 °C, the reaction rate of R‐10 exceeds that of R‐5 by nearly two times. The distinct activities of R‐5 and R‐10 indicate the applicability of the proposed method for rational catalyst tuning. Further comparison of catalytic activity with monometallic catalysts is shown in Figure S10 (Supporting Information) where reaction rate of Pt–Ni nanoparticles is compared to Pt and Ni. Characterization data of ex‐solved particles are listed in Table S2 (Supporting Information) and the reaction rate was normalized by the total surface area of the ex‐solved particles. Pt–Ni exhibits higher catalytic performance compared to monometallic catalysts stating the value of alloy composition control. Lastly, the ex‐solved catalyst was compared with the commercially synthesized catalyst in order to confirm its catalytic feasibility (Figure S11, Supporting Information).

Two methods were used to assess the stability of ex‐solved nanoparticles. Figure S12 (Supporting Information) shows the thermal stability of ex‐solved and impregnated Pt–Ni nanoparticles in comparison to conventionally synthesized catalysts. Both particles were exposed to reducing conditions of 4% H_2_ at 800 °C for 10 h. Negligible size change was observed for ex‐solved particles from 9.3±1.5 to 9.6±1.3 nm in diameter where impregnated particles aggregated, leading to an increase in size from 10.4±2.7 to 16.2±9.1 nm. The stability of the synthesized nanoparticles was also evaluated using TEM image analysis and by a comparison of the catalyst activity during 10 cycles. As shown in Figure S13 (Supporting Information), the particle size distribution was measured by means of TEM before and after three reaction cycles. The particle size increased slightly from 3.1 to 3.6 nm in terms of the diameter but still maintained a desirable size as a nanocatalyst. Considering the fact that the reaction condition is a reducing atmosphere and continuous ex‐solution can occur, such a slight increase in the particle size will be negligible. Moreover, the light‐off curves during 10 cycles of RWGS plotted in Figure S14 (Supporting Information) rather improved every cycle, thus highlighting the stability of the catalyst synthesized through the proposed method. The slight increase in activity is attributed to continuous metal diffusion and corresponding particle growth in the reducing reaction conditions. Table S3 in the Supporting Information shows the characterization data of ex‐solved nanoparticles before and after 10 cycles of RWGS.

### 2.5 Reproducibility of Ex‐Solved Particles

Particle aggregation and a loss of the active surface area during high‐temperature reactions were chronic issues of the supported nanoparticles. Particle reproducibility through a redox heat treatment cycle is another unique advantage of ex‐solved particles. Because ex‐solved particles are socketed in the grain boundaries, by oxidizing the sample with adequate conditions, it is possible to in‐diffuse the metal back into the grain boundary. In the same sense, it is possible to regenerate particles through a subsequent reduction step, allowing perfect reproducibility of degraded or aggregated nanoparticles. We verify this unique property of ex‐solved particles by two means. SEM images of the ex‐solved and oxidized samples are shown in Figure S15 in the Supporting Information. After oxidation at 800 °C for 10 h, no particles were observed on the sample surface. When the sample was again reduced with identical conditions, the particles were ex‐solved again. This phenomenon is also evidenced by the distinct catalytic performances. CO oxidation was performed with each sample after a sequential redox cycle. Figure 4f,g shows the light‐off‐curve for CO oxidation and the temperature at 10% conversion for the samples after each redox cycle. Contrary to the activity of the oxidized samples, which is very low, the activity of the reduced samples was decent and consistent regardless of the number of redox cycles. The high activity of the reduced samples stems from the ex‐solved alloy nanoparticles on the CeO_2_ surface. This distinct catalytic activity is a testament to the reproducibility of ex‐solved particles.

## 3 Conclusion

In this contribution, we designed an alloy system with exceptional tunability on CeO_2_ with heterogeneous doping method for the first time. Through manipulation of the reduction conditions, particles with various dispersion characteristics and compositions were acquired. We interpreted the unique behavior of the various alloy compositions through the distinct kinetics of Pt and Ni in the CeO_2_ grain boundaries by virtue of ToF‐SIMS analysis. Eventually, Pt–Ni alloy nanoparticles exhibit unmatched activity than that of the Ni catalyst in the reverse water gas shift reaction exceeding over 170 times. Anchorage of the synthesized particles hindered particle agglomeration and led to excellent stability during the reaction. Moreover, the close connection between the ex‐solved particles and the grain boundaries allowed reversible particle evolution, ensuring solid reproducibility of the anchored particles. We believe that our systematic blueprint for multi‐component catalyst design offers rational guidance, shedding light on the novel methodology for supported catalyst synthesis to yield robustness in various reactions including solid oxide cells, metal–air batteries, and automobile converters.

## 4 Experimental Section

### Material Preparation

The metal source was deposited by means of magnetron sputtering at a DC power of 100 W in a 10 mTorr Ar atmosphere on a *γ*‐Al_2_O_3_ substrate 10 × 10 × 0.5 mm^3^ in size. Pt and Ni were sputtered layer by layer for two and six seconds, respectively, for a total of 20 layers (10 layers each), after which annealing took place at 800 °C for 5 h in air. The total thickness of the annealed layer was close to 100 nm. Annealing of the metal layer was done to prevent the structural breakdown of CeO_2_ during the subsequent oxidation process. After the deposition and annealing of the metal, 250‐nm‐thick CeO_2_ was deposited by PLD from an oxide target of CeO_2−_ *_*δ*_*. The CeO_2−_ *_*δ*_* target was prepared by a combined EDTA (ethylenediaminetetraacetic acid)‐citrate complexing method. A Ce(NO_3_)_3_·6H_2_O precursor, EDTA, and a citrate agent were dissolved in D.I. water and NH_4_OH was added to ensure a pH level of 9. After homogenization, the solution was kept at 80 °C for gelation. The formed gel was fired at 450 °C for 4 h and calcined at 700 °C for 6 h. The resulting powder was pressed at a pressure of 100 MPa and the pressed target was sintered at 1400 °C for 6 h. A 248 nm wavelength laser with energy of 320 mJ was used for the deposition of the CeO_2_ film, with pulsing done at a 10 Hz frequency (Coherent COMPexPro 201F KrF excimer laser, United States). The deposition temperature and atmosphere were 500 °C and 10 mTorr O_2_ and 500 °C and 150 mTorr O_2_ for the dense and columnar CeO_2_ layer, respectively. After the deposition of CeO_2_, the sample was oxidized for heterogeneous doping of the metal source. Metal was diffused in the CeO_2_ support grain boundaries through oxidation at 700 °C for 10 h under Ar‐balanced oxygen atmosphere (79% Ar, 21% O_2_, 100 mL min^−1^). The metal nanoparticles were then ex‐solved to the surface through a subsequent reduction process. Reduction was accomplished using Ar‐balanced 4% H_2_ under different conditions of 600, 700, and 800 °C for 10 h and 5, 10, 20, and 40 h at 700 °C for particle size and alloy composition control. All annealing processes were done in a tube furnace.

### Physical and Chemical Characterization

The crystallinity and grain size of the synthesized CeO_2_ film were determined through X‐ray diffraction (XRD). Due to the large peak of the Al_2_O_3_ substrate, both 2theta and theta‐2theta scans were taken for a precise analysis. The size and distribution of the synthesized nanoparticles and the grain size were observed through FE‐SEM (SU8230, Japan). An acceleration voltage of 5 kV and an emission current of 5 µA were used to optimize the resolution and minimize charging due to the low conductivity of CeO_2_. A chemical analysis of the nanoparticles was conducted through STEM EDS (Titan cubed G2 60–300, United States) using a ChemiSTEM EDX system based on a 4 windowless Silicon Drift Detectors (Super X). A minimum of 6 to a maximum of 12 particles were used for each sample to estimate alloy composition, and the deviation is shown as error bars. Sampling for the TEM analysis was done with a dual‐beam FIB (Helios NanoLab, United States) device. Further analysis using HRTEM was conducted for an FFT analysis. Concentration profiles of the diffused metal source in the grain boundaries of CeO_2_ were observed using ToF‐SIMS (TOF‐SIMS5, Germany). In this case, 30 keV Bi^−^ ions scanned an area of 100×100 µm^2^ and generated secondary ions while 2 keV Cs ion sputtering was used to etch an area of 300×300 µm^2^. The signals of CeO^−^, ^194^Pt^−^, and Ni^−^ were used for an elemental analysis of Ce, Pt, and Ni, respectively. After obtaining the depth profile, conversion of the sputter time to the depth was done by sputtering CeO_2_ film with a known depth. The signals of all elements were normalized according to the CeO^−^ count in order to compare the ionic current accurately between every sample. The interface between CeO_2_ and the Pt–Ni metal layer was set at the point where the CeO^−^ and ^194^Pt^−^ signals were converted.

### Catalytic Activity Measurement

The reverse water gas‐shift (RWGS) reaction was conducted in a fixed bed reactor (1/2 inch diameter quartz reactor) using eight 10 × 10 mm^2^ samples (Pt–Ni alloy nanoparticles ex‐solved on columnar CeO_2_ support (Pt–Ni/CeO_2_)) stacked on quartz wool. Pt–Ni nanoparticles with different compositions were synthesized through oxidation at 700 °C for 10 h and subsequent reduction at 700 °C for 5 and 10 h, respectively. Ex‐solved Pt and Ni nanoparticles were synthesized by deposition of CeO_2_ on Pt and Ni, respectively, with oxidation and reduction at 700 °C for 10 h. The temperature of the catalyst was measured using a K‐type thermocouple. A mixture of Ar‐balanced CH_4_ and H_2_ (CH_4_:H_2_ = 1 : 4) was used with a total flow rate of 50 mL min^−1^ and the measurements were taken from 200 to 700 °C at a heating rate of 5 °C min^−1^. Gas analysis was conducted via mass spectrometry (Pfeiffer Vacuum GSD320, Germany) for a real‐time analysis of the produced gas. The performance of each catalyst was normalized by the surface area of the catalyst. The performance toward CO oxidation was measured using an identical system. For the reactant, a mixture of Ar‐balanced CO and O_2_ (CO:O_2_ = 1:4) was used with a total flow rate of 50 mL min^−1^. The analysis took place within the temperature range of 100–300 °C at a heating rate of 5 °C min^−1^.

## Conflict of Interest

The authors declare no conflict of interest.

## Supporting information

Supporting Information

## Data Availability

Research data are not shared.
