# Supplementary material for: Synthesis of Highly Tunable Alloy Nanocatalyst through Heterogeneous Doping Method
Source: Adv Sci (Weinh). 2022 Dec 12;10(5):2204693. doi: 10.1002/advs.202204693 (PMC9929244; doi:10.1002/advs.202204693)
Supplement: Supplementary file 1 — Supporting Information [file ADVS-10-2204693-s001.pdf]

ADVANCED SCIENCE
Open Access

Supporting Information

for *Adv. Sci.*, DOI 10.1002/advs.202204693

Synthesis of Highly Tunable Alloy Nanocatalyst through Heterogeneous Doping Method

*Yong Beom Kim, Seunghyun Kim, Jinwook Kim, Jun Kyu Kim, Seung Jin Jeong, DongHwan Oh and WooChul Jung**

Supporting information

Synthesis of Highly Tunable Alloy Nanocatalyst through Heterogeneous Doping Method

Yong Beom Kim[a,1], *Seunghyun Kim*[a,1], *Jinwook Kim*[a], *Jun Kyu Kim*[a,b], *Seung Jin Jeong*[a], *DongHwan Oh*[a] *and WooChul Jung*[*]

a. Department of Materials Science and Engineering, Korea Advanced Institute of Science and Technology (KAIST), 291 Daehak-ro, Yuseong-gu Daejeon 34141, Republic of Korea

b. Current address: Samsung Advanced Institute of Technology (SAIT), 130 Samsung-ro, Yeongtonggu Suwon 16678, Republic of Korea

E-mail: wcjung@kaist.ac.kr (W. Jung)

1. These authors equally contributed to this work.

Table of contents

Figure S1. X-ray photoelectron spectroscopy (XPS) analysis of Pt and Ni diffused in the grain boundaries after the oxidation at 700°C for 10 hours.

Figure S2. X-ray diffraction (XRD) pattern of as-dep (CeO_2/Pt-Ni), oxidized (o-CeO_2/Pt-Ni), and reduced (r-CeO_2/Pt-Ni) sample. Under lines are reference peaks of CeO_2 (JCPDS 34-0394).

Figure S3. XRD pattern and full-width-half-maximum values for three representative peaks for grain size definition of dense CeO_2.

Figure S4. XRD pattern comparison of Pt and Pt-Ni deposited on Al_2O_3 substrate after annealing at 800°C for 5 hours.

Figure S5. High resolution TEM image and corresponding FFT (Fast Fourier Transform) analysis of synthesized CeO_2 after grain boundary ex-solution.

Figure S6. Surface SEM images of ex-solved Pt-Ni nanoparticles on CeO_2 films in terms of reduction temperature (600, 700, 800 °C).

Figure S7. Cross-sectional view of synthesized CeO_2, Pt-Ni stack on Al_2O_3 substrate acquired by FIB.

Figure S8. (a-d) High-angle annular dark-field (HAADF) STEM and energy dispersive X-ray spectroscopy (EDX) mapping images of Pt-Ni alloy nanoparticles in terms of reduction time (5, 10, 20, 40 h). Each sample was oxidized at 700 °C for 10 hours before reduction.

Figure S9. Alloy composition variation of ex-solved Pt-Ni nanoparticles on columnar CeO_2 in terms of reduction time. All oxidation was done at 700 °C for 10 hours and the reduction was done at 700 °C.

Figure S10. Reaction rate of ex-solved Pt-Ni, Pt, and Ni towards reverse water gas shift reaction normalized by surface area.

Figure S11. Light-off-curve (left) and Arrhenius plot (right) of ex-solved Pt-Ni and commercial Ni catalyst towards reverse-water-gas-shift reaction.

Figure S12. SEM images of Pt-Ni nanoparticles synthesized through heterogeneous doping method and impregnation, before and after aging at 800°C for 10 hours (4% H_2).

Figure S13. TEM image and particle distribution (a) before and (b) after 3 cycles of RWGS

reaction.

Figure S14. (a) CO_2 conversion and (b) CO yield in terms of temperature during 7 additional cycles of RWGS reaction.

Figure S15. SEM image of Pt-Ni/CeO_2 (a) after reduction at 800 °C, (b) after re-oxidation of reduced sample at 800 °C, and (c) after re-reduction of the re-oxidized sample at 800 °C.

Table S1. Average particle size, particle density, and composition of Pt-Ni nanoparticle ex-solved on columnar CeO_2 used in catalyst measurement

Table S2. Average particle size, particle density, and composition of the ex-solved nanoparticles of Pt-Ni, Ni, and Pt catalyst used in catalyst measurement.

Table S3. Particle size, density and alloy composition of the Pt-Ni nanoparticles before and after RWGS reaction.

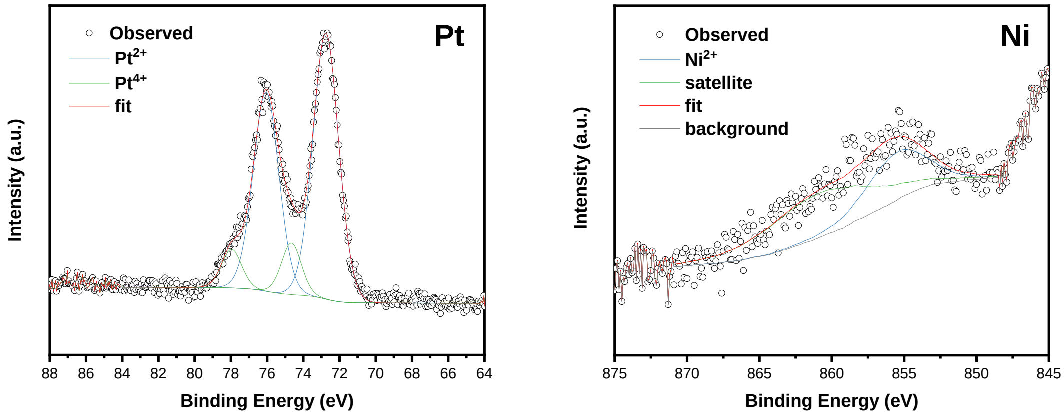

Figure S1. X-ray photoelectron spectroscopy (XPS) analysis of Pt and Ni diffused in the grain boundaries after the oxidation at 700°C for 10 hours.

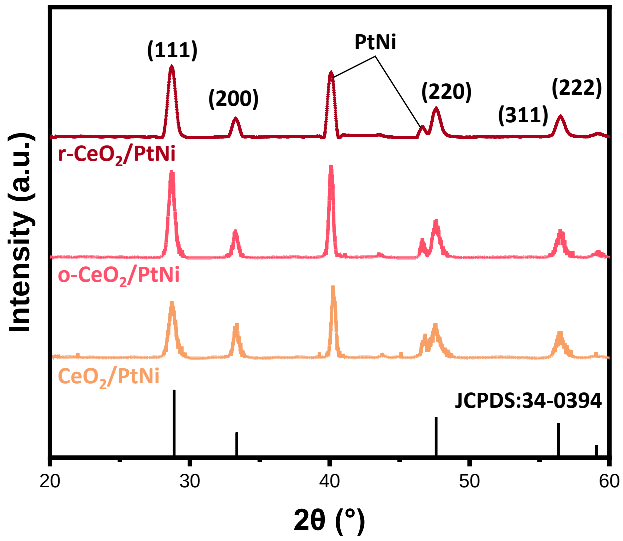

Figure S2. X-ray diffraction (XRD) pattern of as-dep (CeO_2/Pt-Ni), oxidized (o-CeO_2/Pt-Ni), and reduced (r-CeO_2/Pt-Ni) sample. Under lines are reference peaks of CeO_2 (JCPDS 34-0394).

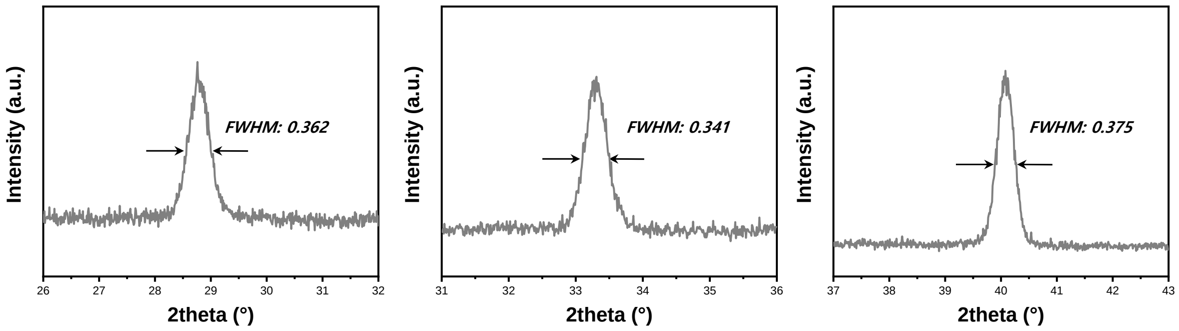

Figure S3. XRD pattern and full-width-half-maximum values for three representative peaks for grain size definition of dense CeO_2.

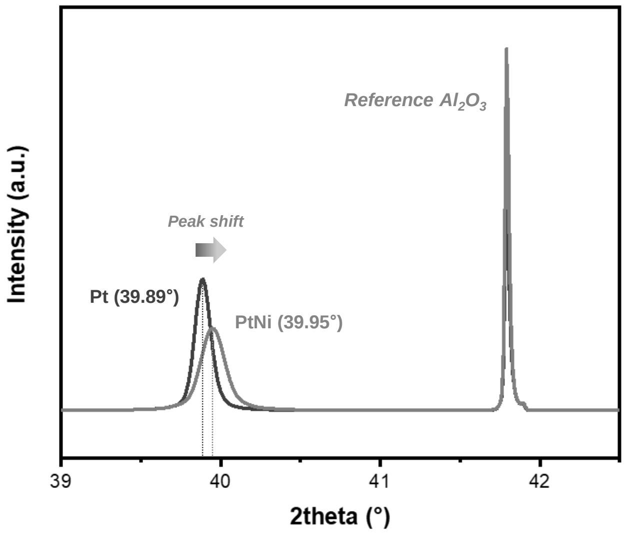

Figure S4. X-ray diffraction (XRD) pattern comparison of Pt and Pt-Ni deposited on Al_2O_3 substrate after annealing at 800°C for 5 hours.

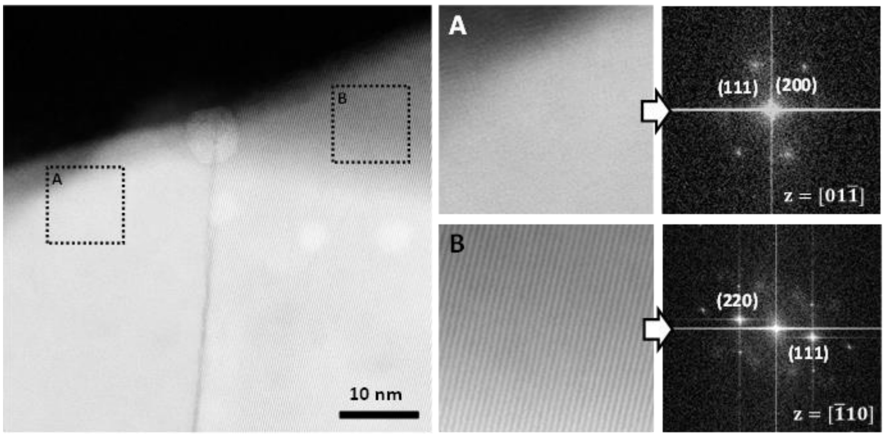

Figure S5. High resolution TEM image and corresponding FFT (Fast Fourier Transform) analysis of synthesized CeO_2 after grain boundary ex-solution.

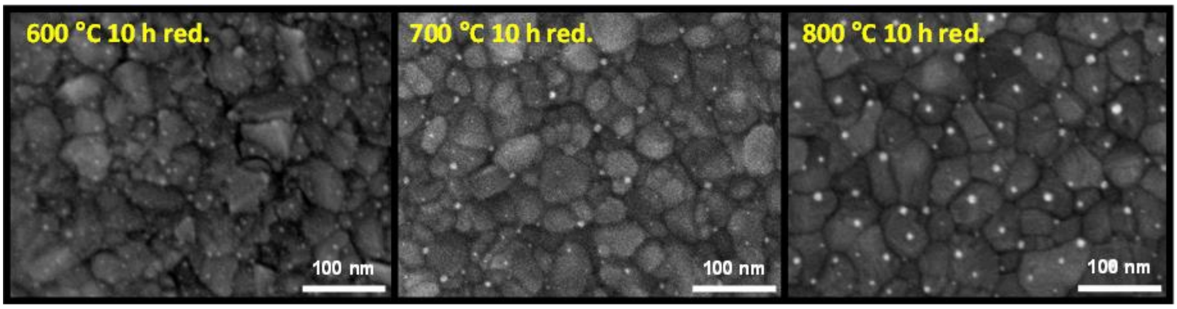

Figure S6. Surface SEM images of ex-solved Pt-Ni nanoparticles on CeO_2 films in terms of reduction temperature (600, 700, 800 °C).

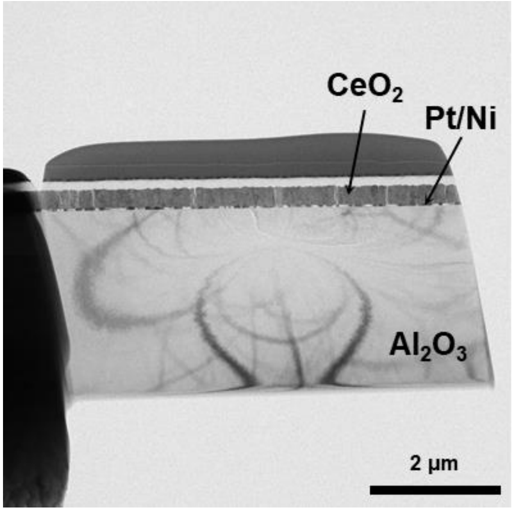

Figure S7. Cross-sectional view of synthesized CeO_2, Pt-Ni stack on Al_2O_3 substrate acquired by FIB.

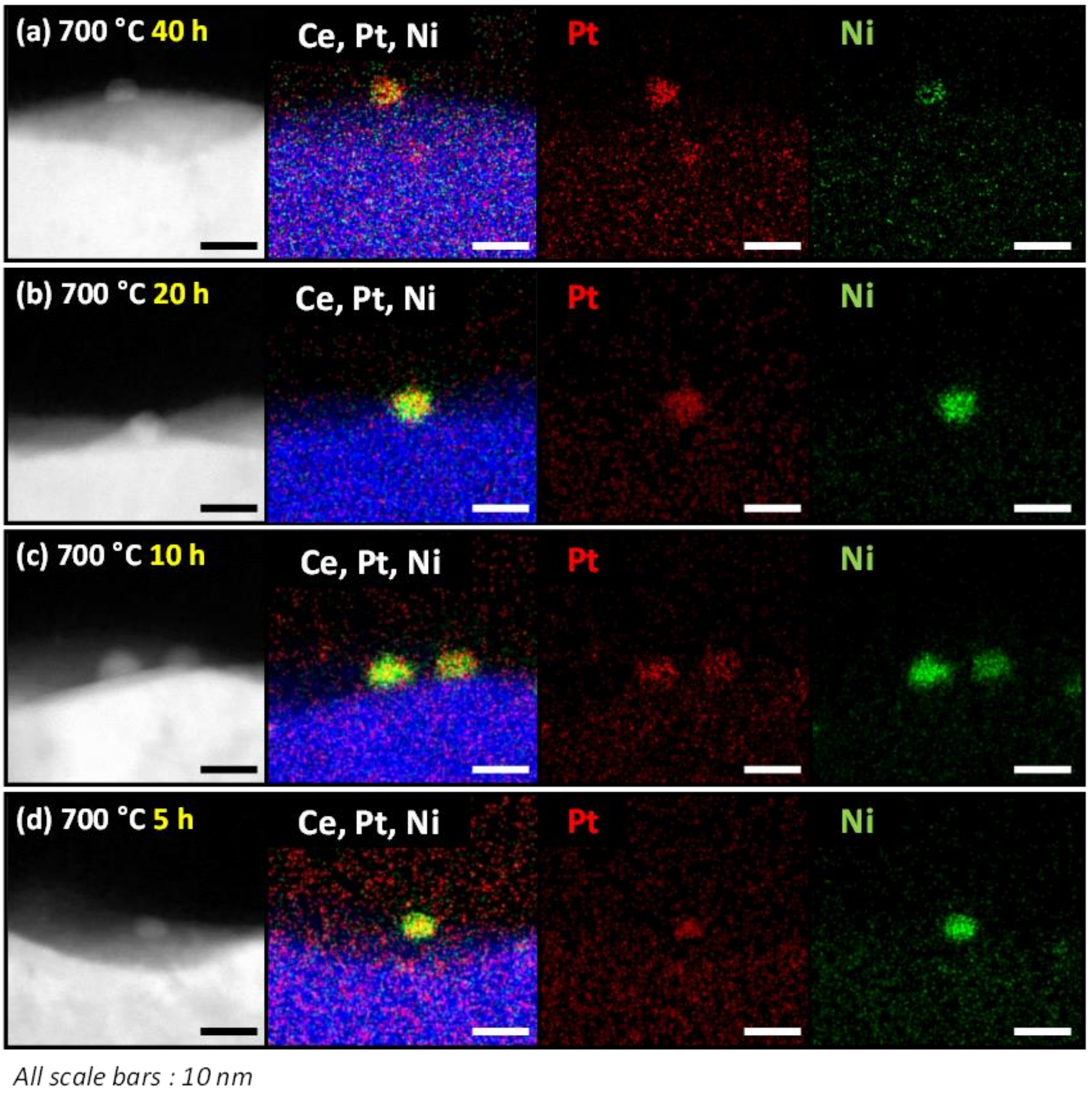

Figure S8. (a-d) High-angle annular dark-field (HAADF) STEM and energy dispersive X-ray spectroscopy (EDX) mapping images of Pt-Ni alloy nanoparticles in terms of reduction time (5, 10, 20, 40 h). Each sample was oxidized at 700 °C for 10 hours before reduction.

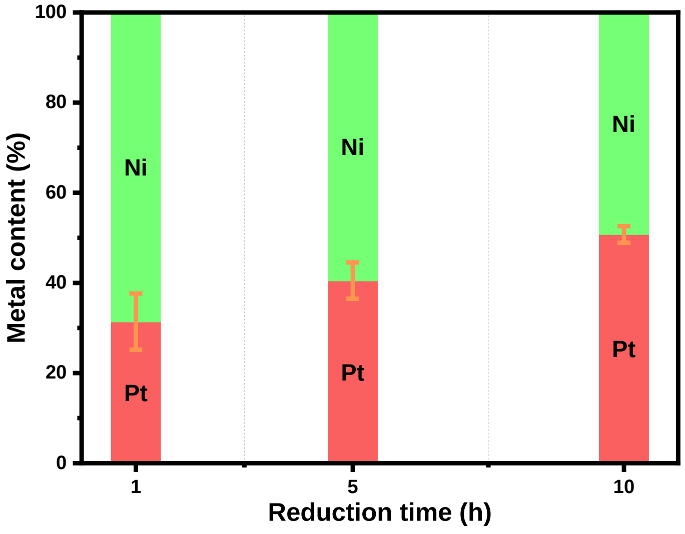

Figure S9. Alloy composition variation of ex-solved Pt-Ni nanoparticles on columnar CeO_2 in terms of reduction time. All oxidation was done at 700 °C for 10 hours and the reduction was done at 700 °C.

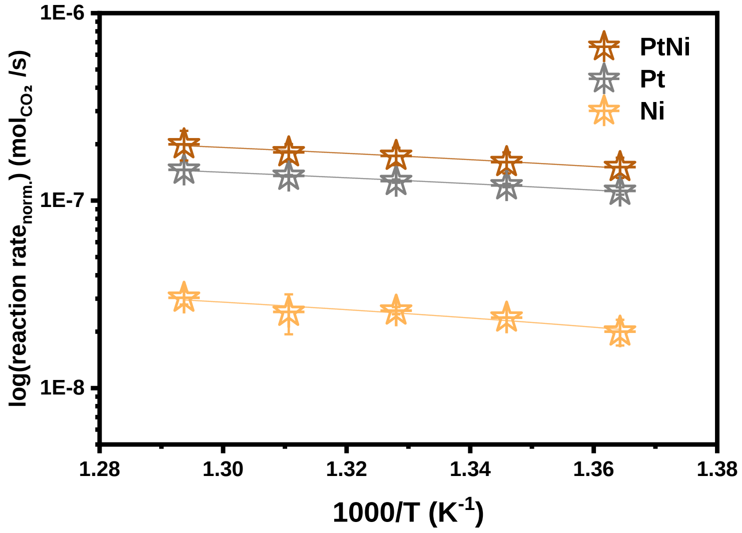

Figure S10. Reaction rate of ex-solved Pt-Ni, Pt, and Ni towards reverse water gas shift reaction normalized by surface area.

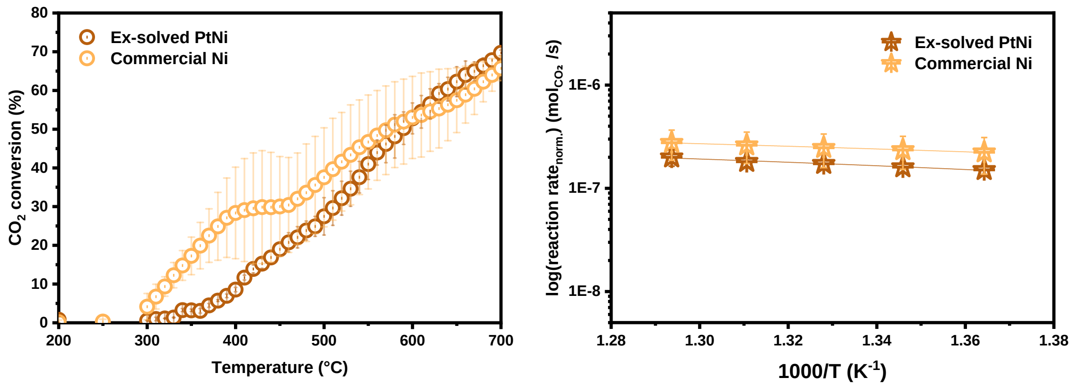

Figure S11. Light-off-curve (left) and Arrhenius plot (right) of ex-solved Pt-Ni and commercial Ni catalyst towards reverse-water-gas-shift reaction.

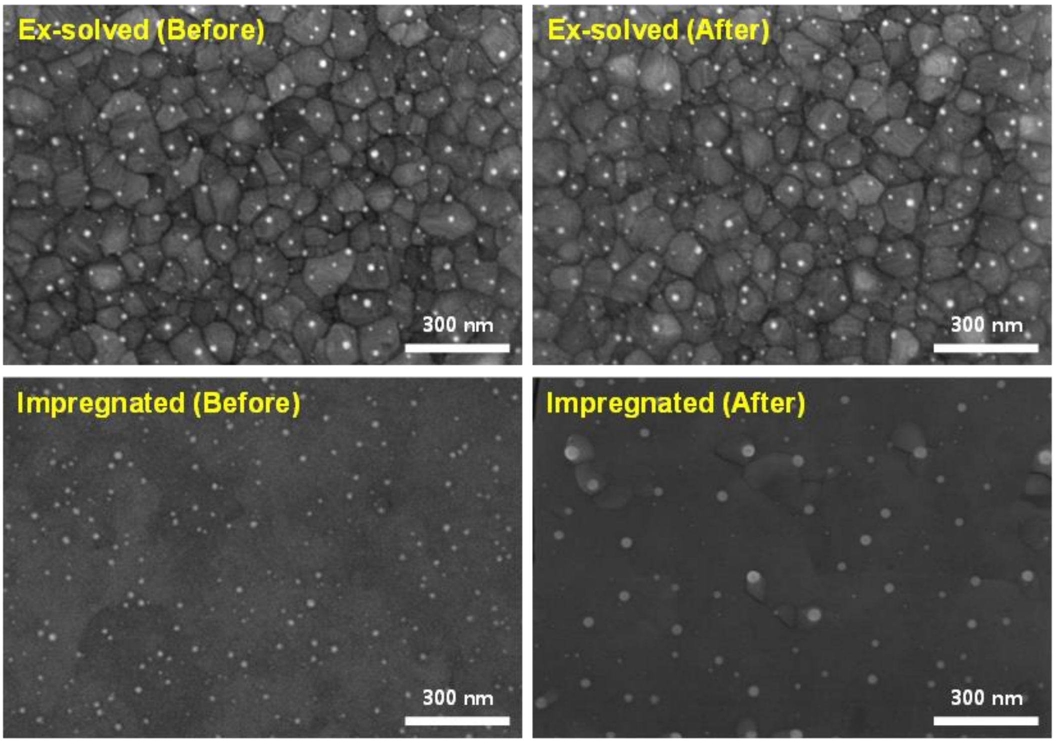

Figure S12. SEM images of Pt-Ni nanoparticles synthesized through heterogeneous doping method and impregnation, before and after aging at 800°C for 10 hours (4% H_2).

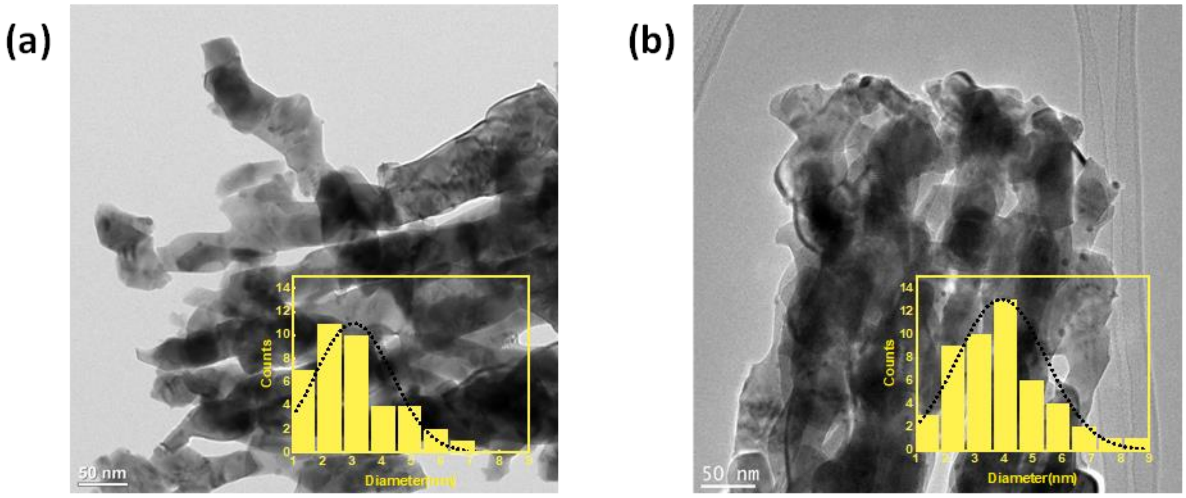

Figure S13. TEM image and particle distribution (a) before and (b) after 3 cycles of RWGS reaction.

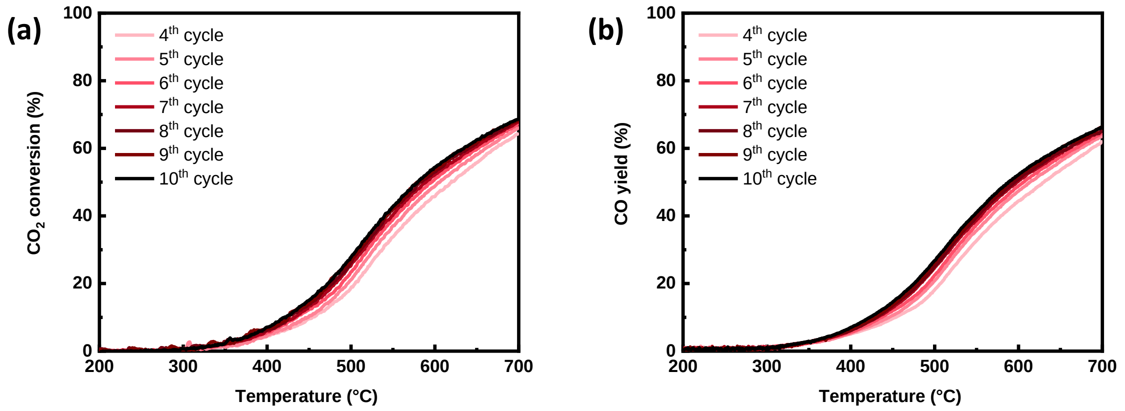

Figure S14. (a) CO_2 conversion and (b) CO yield in terms of temperature during 7 additional cycles of RWGS reaction.

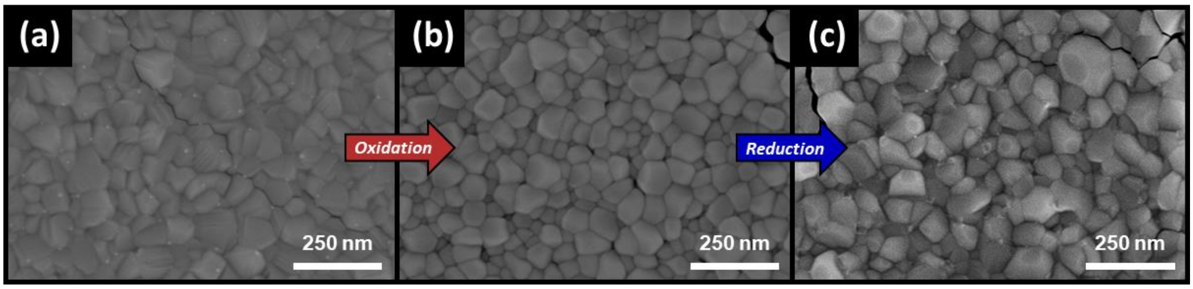

Figure S15. SEM image of Pt-Ni/CeO_2 (a) after reduction at 800 °C, (b) after re-oxidation of reduced sample at 800 °C, and (c) after re-reduction of the re-oxidized sample at 800 °C.

Reduction	Diameter (nm)	Density (#/nm^2)	Composition (%Pt)
700 °C 5 h	3.2±0.3	2.5×10^{-3}	40.5±4.0
700 °C 10 h	3.5±0.4	2.5×10^{-3}	50.8±1.9

Table S1. Average particle size, particle density, and composition of Pt-Ni nanoparticle ex-solved on columnar CeO_2 used in catalyst measurement

	Diameter (nm)	Density (#/nm^2)	Composition (%Pt)
Pt-Ni	3.5±0.4	2.5×10^{-3}	50.8±1.9
Ni	3.3±0.9	2.5×10^{-3}	0
Pt	3.0±1.4	2.5×10^{-3}	100

Table S2. Average particle size, particle density, and composition of the ex-solved nanoparticles of Pt-Ni, Ni, and Pt catalyst used in catalyst measurement.

	Diameter (nm)	Density (#/nm^2)	Composition (%Pt)
Before reaction	3.1±1.3	2.2×10^{-3}	50.2±2.2
After reaction	3.7±1.7	2.3×10^{-3}	55.4±3.1

Table S3. Particle size, density and alloy composition of the Pt-Ni nanoparticles before and after RWGS reaction.

Fundamentals and the derivation process of precipitation kinetics and corresponding flux

Classical precipitation kinetics were employed for the interpretation of grain boundary ex-solution.[1,2] Here we investigate the two ideal cases of precipitation; diffusion controlled kinetics and interface reaction controlled kinetics.

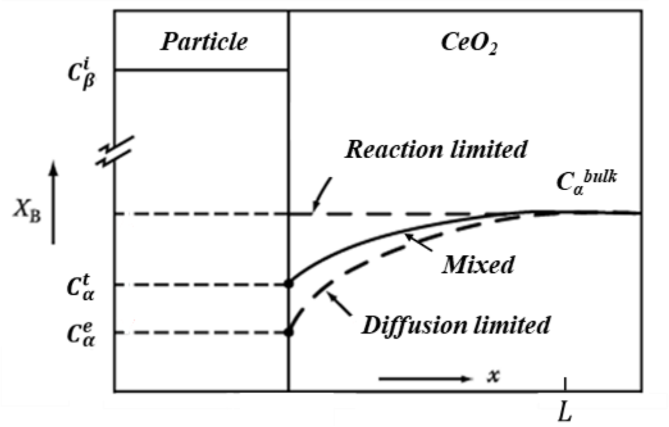

$$\boldsymbol{C_\alpha^t} = Concentration\ of\ B\ near\ the\ surface\ at\ time\ t$$
$$\boldsymbol{C_\alpha^e} = Concentration\ of\ B\ in\ \alpha\ at\ equilibrium$$
$$\boldsymbol{C_\alpha^{bulk}} = Concentration\ of\ B\ inside\ the\ \alpha\ bulk$$
$$\boldsymbol{D} = Diffusion\ coefficient$$
$$\boldsymbol{M} = Interface\ mobility$$
$$\boldsymbol{a} = Length\ of\ the\ perpendicular\ axis$$

Heterogenous precipitation can be seen as a two-step process; the diffusion of the element towards the interface and the interface reaction into the secondary phase. Concerning two specific cases where the total process possess diffusion limited and reaction limited kinetics, the flux of the total process can converge to the following equations. The formula of each diffusive flux and interfacial reaction flux is stated as below.

Reaction flux

$$\boldsymbol{J_i = -MC_\alpha^t k_\beta T \nabla \mu^B = -MC_\alpha^t k_\beta T \left(\frac{\Delta\mu^B}{a}\right) = -MC_\alpha^t k_\beta T \left(\frac{\mu_\alpha^B - \mu_\beta^B}{a}\right)}$$

$$\boldsymbol{= -\frac{MC_\alpha^t k_\beta T}{a}\left(k_B T ln\left(\frac{C_\alpha^t}{C_\alpha^e}\right) - k_B T ln\left(\frac{C_\beta^t}{C_\beta^e}\right)\right) \approx -\frac{MC_\alpha^t k_\beta T}{a} ln\left(\frac{C_\alpha^t}{C_\alpha^e}\right)}$$

Diffusive flux

$$\boldsymbol{J_\alpha = -D\left(\frac{\Delta\mu}{\Delta x}\right) \approx -D\left(\frac{C_\alpha^{bulk} - C_\alpha^t}{L}\right)}$$

On the other hand, when the surface reaction of particle ex-solution is slower than the diffusion of metal, the total flux will equal to the flux of interfacial reaction.

$$\boldsymbol{J_{total} \approx J_i \approx -\frac{MC_\alpha^t k_\beta T}{a} ln\left(\frac{C_\alpha^t}{C_\alpha^e}\right)} \qquad \text{(Equation S1)}$$

If the diffusion of the metal source is slower than the surface reaction, the total flux of particle ex-solution equals that of metal diffusion as given below.

$$\boldsymbol{J_{total} \approx J_\alpha \approx -D\left(\frac{C_\alpha^{bulk} - C_\alpha^t}{L}\right)} \qquad \text{(Equation S2)}$$

[1] D. A. Porter , Easterling, K. E.,, Sherif, Mohamed Y.,, *Phase Transformations in Metals and Alloys.*, **2009**.

[2] W. Preis, W. Sitte, *J. Appl. Phys.* **2005**, *97*, DOI 10.1063/1.1882770.
